# Editorial for the Special Issue “Benefits of Supplementation with L-arginine, Citrulline and Watermelon on Vascular and Metabolic Health”

**DOI:** 10.3390/nu15061491

**Published:** 2023-03-20

**Authors:** Arturo Figueroa, Alexei Wong

**Affiliations:** 1 Department of Kinesiology and Sport Management, Texas Tech University, Lubbock, TX 79409, USA; 2 Department of Health and Human Performance, Marymount University, Arlington, VA 22207, USA

## 1. Introduction

The endothelium is crucial in controlling blood pressure and preventing cardiovascular diseases. In endothelial cells, adequate arginine (ARG) availability for nitric oxide (NO) synthesis is essential to prevent arterial stiffening and hypertension. NO is a potent vasodilator and regulator of blood pressure and organ blood flow. The amino acid ARG is the only substrate for endothelial NO synthase to produce NO. However, in some conditions, such as aging, menopause, hypertension, and obesity, ARG becomes the substrate for an overactive arginase, leading to ARG deficiency and endothelial dysfunction. Previous studies have used ARG supplementation to improve vascular function with mixed results. Moreover, oral citrulline (CIT) can be a precursor of ARG and NO in the kidneys and endothelium, respectively. Given arginase does not catabolize CIT, oral CIT supplementation efficiently increases circulating ARG availability for NO synthesis [1]. Watermelon is a natural source of CIT and ARG, with the rind containing more CIT than the flesh [2]. CIT supplementation, synthetic or from watermelons, improved plasma ARG and NO levels, peripheral arterial stiffness, and blood pressure in middle-aged and older adults [1], demonstrating vascular protective effects. However, researchers know little about the CIT and watermelon supplementation effects on blood flow and endothelial function, with flow-mediated dilation (FMD) assessments. This Special Issue presents two articles on the impact of watermelon on vascular functions. Furthermore, two additional articles provide evidence of the benefits of synthetic CIT supplementation on FMD and skeletal muscle function in postmenopausal women.

## 2. The Watermelon Studies

In a randomized, double-blind crossover study, Fujie et al. [3] investigated the acute effects of wild watermelon-extracted juice ingestion on central and peripheral arterial stiffness and blood flow, and plasma NO levels in healthy young females. The results showed that increased NO bioavailability with wild watermelon juice improved lower-limb arterial stiffness (femoral-ankle pulse wave velocity) and blood flow of the posterior tibial artery. Interestingly, acute watermelon ingestion did not affect aortic stiffness and carotid artery blood flow, indicating that watermelon affects peripheral but not central arteries in young healthy women. These findings establish the framework for future studies of chronic watermelon intake as a preventative therapeutic strategy against peripheral vascular dysfunction in high-risk populations.

Volino-Souza et al. [4] reviewed the effects of watermelon ingestion on vascular health and proposed utilizing food science and technology to improve its efficacy. The authors extensively described CIT properties and mechanisms of vascular dysfunction. Additionally, they illustrated the effects of watermelon ingestion on the main components of vascular health, including endothelial function, arterial stiffness, aortic hemodynamics, blood pressure, and vascular biomarkers. The studies discussed by Volino-Souza et al. found that watermelon supplementation does not affect aortic stiffness but reduces peripheral arterial stiffness and indices of wave reflection in postmenopausal women. In contrast, recent studies failed to improve endothelial function (i.e., FMD) following watermelon juice supplementation in healthy normotensive adults. This ineffectiveness may be attributed to the participants’ good health. Moreover, the amount of CIT in watermelon products may be a limiting factor for beneficial vascular effects [4]. The authors proposed using food technologies such as microencapsulation to deliver higher CIT doses in a lower watermelon volume. 

## 3. The Citrulline Studies

In this Special Issue, two original research manuscripts focused on the effects of CIT on endothelial function assessed by FMD in postmenopausal women with hypertension. Postmenopausal women experience reduced ARG availability which leads to endothelial dysfunction. Endothelial dysfunction precedes arterial stiffness and hypertension [5]. Maharaj et al. examined the results of 4 weeks of CIT on brachial artery FMD, plasma ARG levels, and aortic blood pressure [6]. The authors found that CIT supplementation improved these vascular parameters. In addition to its vascular effects, CIT supplementation with exercise training increased muscle mass or strength in older adults [7,8]. Previous studies did not use resistance training for exercise. However, Kang et al. [9] employed resistance training for investigating the effects of CIT supplementation on superficial femoral FMD, leg lean mass measured by dual-energy X-ray absorptiometry, and leg muscle strength. They randomized participants to CIT or placebo for 8 weeks. For the first 4 weeks, they were on supplementation alone. Then, during the remaining 4 weeks, both groups performed slow-velocity low-intensity resistance training (SVLIRT) for the leg muscles. Kang et al. proved that CIT supplementation alone significantly increases FMD without improvements in leg muscle mass and strength. Interestingly, the combination of CIT and SVLIRT improved leg FMD, lean mass, and muscle strength compared to placebo and SVLIRT. These two manuscripts showed that CIT supplementation significantly improves the endothelial function of arm and leg arteries and, when combined with resistance training, has additive effects on the muscles of the trained limb. Future studies should evaluate the integration of CIT supplementation and resistance training to improve vascular and muscular function in populations with endothelial dysfunction and muscle abnormalities, such as sarcopenia, dynapenia, and exercise intolerance. 

## 4. Conclusions

Endothelial dysfunction can impair arterial structure and function, leading to wall stiffness, atherosclerosis, and hypertension [5]. These pathophysiological conditions increase the risk of cardiovascular morbidity and mortality by restricting organ blood flow and damaging arterial and ventricular walls [5]. Dietary ARG precursors, such as watermelon and CIT, may significantly impact ameliorating vascular dysfunction, particularly in middle-aged and older adults with cardiometabolic risk factors or diseases. However, most clinical trials on the effects of CIT and watermelon were performed in healthy, overweight, obese, and hypertensive individuals. This Special Issue highlights the potential vascular and muscular benefits of watermelon, CIT, or CIT combined with resistance training in individuals with cardiometabolic risk factors or diseases.
